# A Literature Review of Biological and Bio-Rational Control Strategies for Slugs: Current Research and Future Prospects

**DOI:** 10.3390/insects12060541

**Published:** 2021-06-10

**Authors:** Archita Barua, Christopher D. Williams, Jenna L. Ross

**Affiliations:** 1 Crop Health and Protection Limited (CHAP), York Biotech Campus, Sand Hutton, York YO41 1LZ, UK; archita.barua@chap-solutions.co.uk; 2 School of Biological and Environmental Sciences, Liverpool John Moores University, Liverpool L3 3AF, UK; C.D.Williams@ljmu.ac.uk; 3 School of Biological Sciences, University of Aberdeen, Aberdeen AB24 3UU, UK

**Keywords:** biocontrol, bio-rational control, molluscs, slugs, integrated pest management, literature review

## Abstract

**Simple Summary:**

Terrestrial molluscs (slugs and snails) pose a major threat to agriculture, causing severe yield losses in a wide range of crops worldwide. The limited number of chemical molluscicides on the market, along with their negative impact on nontarget organisms and the environment, make mollusc control a real concern for growers and farmers. Therefore, the exploration of alternative, effective and eco-friendly control measures has become a dire need. This study focuses on slugs, as opposed to snails, and reviews the literature on three natural enemies of slugs, namely nematodes, carabid beetles and marsh flies, along with various natural products with slug control potential (for example, essential oils), and this study contributes to providing a comprehensive understanding of how slugs can be better controlled by using nonchemical measures. In doing so, this study also draws attention to the limitations of current research and discusses some important future research avenues in order to develop effective nonchemical slug control measures.

**Abstract:**

Terrestrial gastropod molluscs (slugs and snails) (Mollusca: Gastropoda) cause significant crop damage around the world. There is no formal approach for differentiating between slugs and snails; however, an organism is usually considered a slug when there is no external shell, or when the shell is small in comparison to the body, and a snail when there is a large external shell. Although snails are an important pest of many crops, this review focuses on slug pests and their nonchemical control measures. A recent study by the UK Agriculture and Horticulture Development Board concluded that the failure to control slugs could cost the UK agriculture industry over GBP 100 million annually, with similar figures reported around the world. Whilst slugs are mostly controlled using chemical molluscicide products, some actives have come under scrutiny due to their detrimental environmental effects and impact on nontarget organisms. This has resulted in the ban of actives such as methiocarb in the UK and EU, and, more recently, the ban of metaldehyde in the UK. Therefore, there is an urgent need to find alternative and effective nontoxic solutions in the interest of global food security. In this paper, we have integrated extant literature on the three main biological control agents of slugs, namely nematodes, carabid beetles and sciomyzid flies, and various promising bio-rational slug control strategies. The review also highlights current research gaps and indicates some relevant potential future directions towards developing environmentally benign slug control solutions.

## 1. Introduction

Terrestrial gastropod molluscs (slugs and snails) (Mollusca: Gastropoda) are among the most successful animal groups in the terrestrial ecosystem [1,2]. To date, there is no formalised method for differentiating between slugs and snails; however, an organism is usually considered a slug when there is no external shell or when the shell is significantly reduced in comparison to the body size, and a snail when there is a large external shell [2]. All slugs have evolved from snails, and this has occurred multiple times throughout evolution [1]. Although snails are an important pest of many crops, this review mainly focuses on slug pests and their nonchemical control measures. Slugs constitute a serious menace to agricultural production, resulting in significant economic damage to a wide range of crops, including oilseed rape, vegetables, legumes, cereals and fruits [1,2,3]. Damage from slugs is particularly severe in regions with a temperate climate, including parts of Europe (e.g., Ireland, France, the Netherlands and the UK), North and South America, Australia, New Zealand and South Africa [4,5,6,7]. A recent study by the UK Agriculture and Horticulture Development Board concluded that the failure to control slugs could cost the UK agriculture industry over GBP 100 million annually [8]. Similar figures have also been reported in other countries around the world. For instance, in the past few years, grass seed fields in the Willamette Valley of Oregon, USA, have suffered about USD 100 million losses due to infestations of the grey field slug (*Deroceras reticulatum* (Muller) and *Arion* spp., impacting approximately one-fifth of the total value of the industry [9].

In the UK, a total of 42 slug species have been identified [10], with the majority being non-native and able to successfully establish their population [7,10]. Extant control strategies for slugs mostly rely on chemical molluscicide pellets, containing either metaldehyde or iron (ferric) phosphate. However, there have been concerns over metaldehyde due to its impact on nontarget organisms and water systems. Political and scientific debates have been going on for several years in different countries regarding the banning of metaldehyde. In 2018, the Department for Environment, Food and Rural Affairs (Defra) in the UK proposed the ban on the use of metaldehyde, which was later overturned by the High Court in London on technical grounds as the ban was challenged by slug pellet manufacturer Chiltern Farm Chemicals [11]. However, recently, Defra has again announced that the outdoor use of metaldehyde will be banned from March 2022 in the interest of the environment to align with the 25 Year Environmental Plan and Environmental Bill in the UK, and to fulfil the sustainable development goals and for wider food security [12].

Due to the limited chemical toolbox, the necessity for alternative, effective and environmentally benign slug control measures has become ever more essential. Two such environmentally benign strategies are biological control (also referred to as biocontrol) and bio-rational control. Biocontrol promotes the controlling of pests using their natural enemies [13], whereas bio-rational control refers to using products that have been derived from natural sources, e.g., plant extracts [14].

Biocontrol and bio-rational control in Integrated Pest Management (IPM) are not new strategies. Due to the declining availability of conventional pesticides because of growing pest resistance and changes in legislation, the demand for biocontrol and bio-rational control has been on the rise in the past decade. The benefits of using biocontrol and bio-rational control in IPM strategies include not only the reduction in the use of toxic chemicals, but also the provision of an additional market for growers who do not use synthetic pesticides [13]. For example, consumers often show a willingness to pay a premium for products grown organically.

There are a variety of biocontrol agents of slugs, such as mites, spiders, rats, frogs, lizards, centipedes, beetles, millipedes, and flatworms [15]. Despite having a wide range of predators, only a few have attracted research interest. Among invertebrates, nematodes, carabid beetles and sciomyzid flies have been reported as promising biocontrol agents of slugs [16,17,18,19]. In parallel, several bio-rational control measures have also been found to be effective in slug control, which includes, but is not limited to, the use of essential oils (e.g., garlic oil and spearmint oil), plant extracts and caffeine [20,21,22].

This review describes the slug control potential of the three main biocontrol agents of slugs, namely nematodes, carabid beetles and sciomyzid flies, along with various bio-rational control strategies that have been studied by scientists to date. The review has been performed on articles, technical reports, policy briefs, book chapters and conference proceedings.

## 2. Extant Research on Biocontrol and Bio-Rational Control of Slugs

### 2.1. Nematodes as a Biocontrol Agent of Slugs

Nematoda is the second most diverse animal phylum after arthropods residing in a wide range of habitats. Nematodes are recognised as an important biocontrol agent of various insects, mites and molluscs pests of economically important agriculture crops, ornamental plants, and forestry [23,24,25,26,27,28]. Over the past decade, extensive scholarly interest has been devoted towards examining the diversity, ecology and molecular phylogeny of nematodes and their biocontrol efficacy against terrestrial slugs. In these studies, the nematode *Phasmarhabditis hermaphrodita* (Schneider) Andrássy (*Pelodytes hermaphroditus*) has drawn the most attention.

*Phasmarhabditis hermaphrodita* was first found in decaying terrestrial molluscs in 1859 and was suggested as *Pelodytes hermaphroditus* [17]. In the UK, it was isolated from the mantle cavity of an infected slug *D. reticulatum* in 1987 [17]. *P. hermaphrodita* is a soil-dwelling facultative mollusc-parasitic nematode that can complete its life on dead invertebrates and decaying leaf litter [29,30]. *P. hermaphrodita* dauer juvenile larvae infect molluscs by entering through natural openings and then developing to the adult stage and subsequently reproducing. Mortality usually follows within 7–21 days [26,31].

Studies have tested the pathogenicity of *P. hermaphrodita* in slugs from several families—Agriolimacidae, Arionidae, Limacidae, Milacidae and Vagnulidae [17,26,32], with pathogenicity varying across slug families. *P. hermaphrodita* is particularly effective against slugs from the Agriolimacidae family, such as *D. reticulatum* and *D.invadens* Reise, Hutchinson, Schunack and Schlitt. The effectiveness of *P. hermaphrodita* against pestiferous slugs has also been reported in countries outside Europe, including the USA [33]. In California, *P. hermaphrodita* was discovered from three invasive slug species, namely *D. reticulatum*, *Deroceras laeve* Muller and *Lehmannia valentiana* Ferussac, along with two other *Phasmarhabditis* species—*P. papillosa* and *P. californica* [34,35]. The presence of *P. hermaphrodita* in New Zealand was recorded in the native slug, *Athoracophorus bitentaculatus* Quoy and Gaimard, which also represented a new host record for the nematode species [36]. Due to the nematode’s effectiveness against *D. reticulatum* (also known as the ‘Grey field slug’), which is the most pestiferous native slug species found in the UK and Ireland [5,19], *P. hermaphrodita* has been formulated as a commercial bio-molluscicide under the product names, Nemaslug^®^ and SlugTech, by BASF and Dudutech, respectively, and is widely used in Europe for slug control [17,26].

However, there currently exist a number of problems with using *P. hermaphrodita* commercially. First, the nematode is not economically feasible for application in arable crops, due to several barriers such as high production and application costs (approx. GBP 110/ha). Studies have reported that the field-level implementation of *P. hermaphrodita* requires a large volume of water; it has strict storage requirements, and it possesses a short shelf life [7]. Second, the pathogenicity of nematode strains may also be a challenge, with BASF’s Nemaslug^®^ product being cultured over a long period of time (25 years), and the possibility that slugs may develop resistance to this strain [37]. Another problem is that studies have shown that *P. hermaphrodita* may not be effective in parasitising larger hosts such as *Arion lusitanicus* (Mabille) [4,38]. For instance, Grimm [38] studied the effect of *P. hermaphrodita* on adult *D. reticulatum* and three different stages of the pest slug, *A. lusitanicus,* and observed that the nematode successfully controlled *D. reticulatum*; however, against young stages of *A. lusitanicus*, the nematode acted as a feeding inhibitor instead. Further investigation is necessary to understand the reasons behind the lack of susceptibility to *P. hermaphrodita* in some slug species. Lastly, the use of *P. hermaphrodita* in bio-molluscicide products is not approved, for biosecurity reasons in some countries outside of Europe due to limited knowledge on its natural distribution. On the other hand, to some degree, researchers have also tried identifying alternative nematode species that can also show similar or a higher slug efficacy capability compared to *P. hermaphrodita*. To date, there are 15 nominal species in the *Phasmarhabditis* genus, namely: *P. hermaphrodita*; *P. neopapillosa* (Mengert *in* Osche) Andrássy; *P. papillosa* Schneider Andrássy; *P. tawfiki* Azzam; *P. huizhouensis* Huang, Ye, Ren and Zhao; *P. apuliae* Nermuť, Půža and Mráček; *P. bonaquaense* Nermuť, Půža, Mekete and Mráček; *P. californica* Tandingan De Ley, Holovachov, Mc Donnell, Bert, Paine and De Ley; *P. bohemica* Nermuť, Půža, Mekete and Mráček; *P. meridionalis* Ivanova and Spiridonov; *P. safricana* Ross, Pieterse, Malan and Ivanova; *P. circassica* Ivanova, Geraskina and Spiridonov; *P. clausiliiae* Ivanova, Geraskina and Spiridonov; *P. zhejiangensis* Zhang and Liu; *P. kenyaensis* Pieterse, Rowson, Tiedt, Malan, Haukeland and Ross. Of these species, 11 are known to associate with slugs (Table 1), and *P. neopapillosa*, *P. californica* and *P. papillosa* have been observed to demonstrate parasitising preferences for several slug species, including *D. reticulatum*, *D. invadens*, *L. valentiana*, *A. ater*, *A. vulgaris* and *A. hortensis* [39,40,41,42]. Moreover, international surveys have been conducted in Europe [7], Asia [43], North America [44], Australia [45] and Africa [46] to identify whether nematodes from other families are associated with molluscs as definitive hosts. Overall, it has been reported that nematode species belonging to the following eight families—Agfidae, Alaninematidae Alloionematidae, Angiostomatidae, Cosmocercidae, Diplogasteridae, Mermithidae and Rhabditidae used molluscs as definitive hosts [40,46,47,48,49,50,51,52,53,54,55,56,57,58,59,60,61,62,63,64,65].

### 2.2. Sciomyzid Flies as a Biocontrol Agent of Slugs

Flies belonging to the family Sciomyzidae (Order Diptera) are another group of natural enemies of terrestrial gastropods [15,66,67]. The first evidence of larvae of sciomyzid fly preying on gastropod hosts was reported in 1953 [68]. Since then, studies on sciomyzids as a ‘malacophagous group of insects’ have attained much interest among malacologists and evolutionary biologists. Among the natural enemies of terrestrial gastropods, the family Sciomyzidae is one of the most studied groups, and the general interest in the study of Sciomyzidae has led to the development of a dedicated site by a coterie of Sciomyzidae researchers across the world. The website includes a list of publications on Sciomyzidae relating to their key features including feeding behaviour, range of prey/hosts, microhabitat, phenology, and other ecological and behavioural traits [32,67,69,70].

The Sciomyzidae, commonly known as marsh flies, is a small family of acalyptrate flies that contains approximately 550 species in 63 genera globally [71,72]. Of the 550 recorded species, a total of 240 marsh fly species lifecycles have been studied biologically to date, which makes this family the most studied and best-known group of higher Diptera [70,71]. Research has been performed on various aspects of Sciomyzidae in different regions of the world. For instance, [67] pioneered research on all aspects of Sciomyzidae such as their biology, host prey relations, feeding behaviours, phenology and geographic distribution. Vala [73] distributed Sciomyzidae into seven geographic regions, namely Palaearctic, Nearctic, Neotropical, Oriental, Afrotropical, Subantarctic, Australia and Oceanic, and they observed that the diversity of marsh fly species was greater in the first two regions.

Studies on the Sciomyzidae are of particular interest among experts because of their diverse feeding behaviour on a host as the larvae are often neither entirely predaceous nor parasitoid but change during their developmental period. Studies [15,69,71] have demonstrated that their control method (whether predator or parasitoid) is associated with behavioural features such as places of oviposition and pupation, host specificity and seasonal aspects of the species. Vala [73] categorised Sciomyzidae into 16 groups depending on the feeding behaviour and type of food eaten by the larvae. Immature stages display a wide range of feeding behaviours, including predation and parasitism, and it is now well established that Sciomyzidae is the only dipteran family whose larvae possess the malacophagous property [32,70] predating mostly on the snail host [32,74].

The potential of sciomyzid species as biocontrol agents of terrestrial mollusc pests of agriculture was recommended by Khaghaninia [75]. However, records of the efficacy of species of sciomyzids against slugs are very limited. The slug-control potential of Sciomyzidae was first reported in *Tetanocera elata* Fabricius, which was observed to be feeding on several European slug species [69,76]. The larvae of *T. elata* feed as a parasitoid during the first and second instar stage and as a predator in the later stage on slugs. Subsequently, several studies [19,74,77] confirm that larvae of *T. elata* are potential biocontrol agents of slug species belonging to the genera *Deroceras*, *Arion*, *Limacus*, *Limax* and *Tandonia*. Of all the known sciomyzids, only six species, namely, *Tetanocera clara*, *T. plebeja*, *T. valida*, *T. elata*, *Euthycera arcuate* and *E. chaerophylli*, have been documented as slug feeders [67,78]. In addition to *E. arcuate* and *E. chaerophylli*, [1] found two more sciomyzid flies’ larvae, belonging to the genus *Euthycera*, namely, *E. cribrata* (Rondani) and *E. stichospila* (Czerni), to be slug attackers. In addition, [15] conducted a comprehensive review on the biocontrol potential of marsh flies on terrestrial gastropods, where they reviewed the evolution, systematics, diversity, lifecycle, phenology and adaptive specialisation in sciomyzidae that prey on slugs. According to Barker [15]’s observations, species *T. elata* (Fabricius), *T. plebeja* Loew and *T. valida* are host-specific and feed on slugs of the genus *Deroceras* as parasitoids, and the late larval stages of *T. valida* is predaceous.

Table 2 lists different slug-controlling sciomyzid species along with the host slug species studied and the geographical locations in which the observations were made.

The mode of action of sciomyzid flies on slug hosts revealed that, unlike most newly hatched sciomyzid larvae, *T. elata* larvae never crawl in search of their host, remaining motionless near the empty egg membranes. Once a passing slug host is encountered by *T. elata* larvae, the larvae then move to make firm contact with the host and enter the slug’s body via the mantle [6,69]. For the first instar parasitoid larvae, the slug mucus is assumed as the main source of food as the larva increases rapidly in size immediately after attacking a slug host [69]. The slugs remain active for the first few days. However, once the larva attains the third instar, it becomes predaceous, immobilises their prey and starts feeding on the dead tissue of the slug [6]. To immobilise slugs, previous studies suggested that the locomotion activity of slug is inhibited due to the injection of a toxin protein secreted from the salivary glands of the predatory larvae [32]. However, that toxic protein has not been recognised in all slug-controlling sciomyzids [32].

However, there exist some challenges in using *T. elata* as a biocontrol agent for slugs. First, *T. elata* larval stages depend on selected prey slug species for survival, which is a major concern for the mass rearing of *T. elata’s* larva in the laboratory. This also means that *T. elata* cannot be used for controlling all pestiferous slug species. Second, *T. elata* has an irregular distribution which makes this species somewhat difficult to find in the field [19]. In a recent study, [79] observed the habitat requirement of *T. elata* in western Ireland and found that *T. elata* is significantly more abundant in the proximity of hedgerows. A better understanding of the habitat requirement of *T. elata* will help to design a conservation biological control programme and utilise this natural enemy as an alternative to molluscicides. Third, the *T. elata* neonates do not try to find their host by themselves, which implies that the larva comes in contact with their host only when they encounter a host slug accidentally [69]. If the *T. elata* hatchling does not try to find their host or food source by themselves, most of the newly hatched neonates will die from starvation.

### 2.3. Carabid Beetles as a Biocontrol Agent of Slugs

Carabid beetles or ground beetles are a group of insects that belong to the family Carabidae and have been documented as a promising biocontrol agent of many agricultural pests, including slugs [80,81,82,83,84]. Carabid beetles have a global distribution, with a total of 40,000 species reported, of which 2700 species have been found in Europe in diverse habitats, including woodland, gardens, hedgerows and cultivated lands [82]. The general assumption is that carabid beetles are polyphagous predators that feed on any suitable prey [82,85]. Studies on the carabid beetles’ diet (e.g., Ayre [81]) have considered carabids as generalists and opportunistic predators that forage on whatever they encounter. The role of carabid beetles as a natural enemy and their interaction with mollusc pests have been reviewed by many authors [82,83,86].

Studies [86,87,88] have demonstrated that the large carabid beetles in the tribe Cychrini such as *Carabus* spp., *Abax parallelepipedus* Piller and Mitterpacher, and *Pterostichus melanarius* Illiger prey mostly on slugs. Among the *Carabus* spp., *C. nemoralis* predation on slug was first reported by [86] who revealed that preying on gastropods by carabid species is associated with their size. This was confirmed by serological techniques, where Tod [86] recorded a higher quantity of gastropods found in large-sized *Carabus* spp. (e.g., *C. nemoralis* and *C. violaceus*) compared to other species of carabids.

In addition to *C. nemoralis* and *C. violaceus*, Ayre [81] observed that *Cychrus caraboides* is also a mollusc specialist carabid, and it predates on the slug *D. reticulatum*. Furthermore, to assess the effect of beetle size and temperature on the predation of newly hatched *D. reticulatum*, [88] experimented with 21 differently sized carabid species and observed that medium-sized beetles (*Agonum dorsale* and *Agonum fuliginosum*) and large-sized beetles (*P. madidus* and *Harpalus rufipes*) predate more on slugs when the temperature increases. Hatteland [89] revealed that the carabids beetles *P. melanarius*, *P. niger*, *C. nemoralis* and the staphylinid *Staphylinus erythropterus* can all feed on slug eggs and slug hatchlings of *A. lusitanicus*. Digweed [87] studied the feeding preferences of different carabid species on seven terrestrial gastropod prey and found that *Scaphinotus marginatus* (Coleoptera: Carabidae) preyed mostly on the slug *D. reticulatum*. Then, Symondson [90] reviewed five families (Carabidae, Staphylinidae, Lampyridae, Drilidae and Silphidae) of coleopterans as natural enemies of terrestrial gastropods. Drawing on laboratory results, [81] confirmed the predatory potential of several beetles of the carabid genus such as *P. niger* (Schaller), *P. madidus* (Fabricius) and *Abax ater* (Villers) against slug species *D. reticulatum*, *A. subfuscus, A. intermedius, A. circumscriptus* and *A. rufus*. Several other scientists have found that the carabid *P. melanarius* predates on slugs too based on laboratory [89,91,92], miniplots and field studies [83,93].

Although the predatory potential of carabid beetles has been mostly assessed in the context of adult feeding behaviour, the larval stages of carabid beetles (e.g., *P. melanarius*) are also significant predators of the slug [94]. A few studies have reported that carabid beetles also predate on slug eggs [95,96]. In a microplot study, Barker [97] observed that when carabid beetles were introduced, slug species *D. reticulatum* and *A. intermedius* populations were significantly reduced. Similar results were attained by [80], who assessed the potential of *A. parallelepipedus* and *P. madidus* on slug *D. reticulatum* and commented that both species are as effective as the molluscicide methiocarb in controlling slugs. However, Symondson [85] demonstrated that carabid beetle *A. parallelepipedus* is an effective predator of slug *D. reticulatum* in soil surfaces only, but it was less effective in capturing slugs in mature lettuce plants in polythene tunnels.

Table 3 lists different slug-controlling carabid species, along with the host slug species studied and the geographical locations in which the observations were made.

With recent advancements in diagnostic techniques, serological and molecular techniques have been used to assess predator–prey interactions and to analyse the gut contents of predatory beetles [81,84,89]. Earlier, precipitin tests were utilised to identify gastropod protein contents in the carabid and staphylinid predators’ gut. However, it was observed that serological techniques are particularly suitable for sucking predators that suck the juices of prey [81]. To obtain more accurate results, protein-based diagnostic methods have been replaced by DNA-based methods to assess DNA traces of slugs in predators’ guts. Symondson [85], by using a quantitative ELISA test, confirmed that *A. parallelepipedus* predate readily on slugs. Hatteland [89] developed a multiplex polymerase chain reaction (PCR) method to analyse predation by carabid beetles on slugs *A. lusitanicus*, *A. ater* and *A. rufus* by observing gastropod-specific DNA in the predator’s gut. Recently, Reich [84] examined the gut contents of four carabid species, namely *Agonum muelleri*, *Calosoma cancellatum*, *Nebria brevicollis* and *Poecilus laetulus*, in the USA using the qPCR technique, and they revealed the presence of gastropod-specific DNA in three of the carabid species’ guts. Although molecular techniques have been gaining interest as an analytical tool to quantify predator–prey interactions, these methods do not differentiate between predation and scavenging food items [99]. Misinterpretation of the results due to cross-amplification of the target and nontarget DNA is another source of error [89]. An additional shortcoming of using carabid beetles as a biocontrol agent for slugs is that when alternative prey populations are available and are diverse, carabid beetles may move away from slugs and be attracted towards other prey [92]. Further, if the slugs remain below the soil surface, carabid beetles cannot prey on the slugs when they cause damage to seedlings below the ground [105].

### 2.4. Natural Products as Biorational Control Agents of Slugs

Over the past two decades, another parallel research domain has emerged, investigating the potential of using natural products for slug control. As an alternative to synthetic chemicals, plant-derived essential oils (EOs) are increasingly being used as bio-rational products in agriculture. Various scholars [30,106,107,108,109] have argued that essential oils show antimicrobial, acaricidal, insecticidal, molluscicidal and nematicidal properties while demonstrating little or no toxicity to humans and the surrounding environment. The molluscicidal properties of essential oils have been mostly confirmed against terrestrial molluscs [110]. Recently, drawing on laboratory and greenhouse experiments on 13 essential oils, Klein [22] observed that thyme and spearmint oil are lethal to the slug *D. reticulatum*, whereas pine, peppermint, garlic, rosemary, lemongrass, and cinnamon oils show low-to-moderate levels of toxicity against *D. reticulatum*. Klein [22] also detected no phytotoxic effect of these essential oils on plants, which, combined with other advantages, such as the commercial availability of essential oils (unlike the sciomyzid *T. elata*) and no restrictions over its use in agricultural fields in any part of the world (unlike the nematode *P. hermaphrodita*), makes a strong case for essential oils to be included in the management of slug pests.

However, due to their nematicidal and insecticidal properties, the use of essential oils could be harmful to beneficial nematodes and insects. In fact, Barua [30] found that the following essential oils: thyme, cinnamon, clove, garlic, pine and lemongrass, cause mortality in the beneficial nematode species *P. hermaphrodita*, *Steinernema feltiae* and *Heterorhabditis bacteriophora*. In addition to essential oils, scientists have also shown interest in exploring other bio-rational products with slug control capabilities. Some early work cited in [32] observed some ovicidal effects of fungus on slugs and found evidence that *Verticillium chlamydosporium* Goddard prevents the hatching of slug eggs. However, no study followed up on these findings, and hence, research on the diseases caused by microbes on slugs has remained scarce [32].

Although biological control measures such as the use of nematodes, marsh flies and carabid beetles when implemented properly could help in reducing the slug population significantly, it is practically impossible to completely eradicate slugs from agricultural fields. Therefore, it is also important to look for strategies that can enhance crops’ defensive properties against the pest, which are: (a) repellent/barrier properties, i.e., the pest does not come in contact with the crop; (b) irritant properties, i.e., the pest moves away after coming in contact with the crop; and (c) antifeedant properties, i.e., the pest does not feed on the crop [111]. Enhancing the crop’s repellent, irritant and antifeedant properties are useful in implementing the ‘push’ strategy in integrated pest management [112], which refers to the behavioural manipulation of pests through the incorporation of stimuli that make the protected resource unattractive to the pests.

Prior studies have, to a certain extent, examined the repellent and antifeedant properties of various natural products, including essential oils and plant extracts against slugs [20,21,42,113]. Among essential oils, by drawing on laboratory trials, [113] highlighted that the essential oil of Myrrhs (*Commiphora molmol* and *Commiphora guidotti*) possesses repellent properties against molluscs *D. reticulatum* and *A. hortensis*. Similarly, Lindqvist [114] demonstrated that when birch tar oil was sprayed on vegetable pots, it effectively repelled slugs *A. lusitanicus*. However, it was mentioned that to fully understand the effect of birch tar oil on slug management as an effective, economic and environmentally friendly slug repellent, further investigation of the chemical composition of birch tar oil is required [114]. On the other hand, among plant extracts, [20] applied the methanol extracts of the plants *Geranium robertianum*, *Lepidium sativum*, *Origanum vulgare*, *Salvia officinalis*, *Salvia pratensis*, *Saponaria officinalis*, *Thymus vulgaris*, *Trifolium repens* and *Valerianella locusta* on rape seedlings to examine whether the methanol extracts can reduce the feeding of the slug *A. lusitanicus*. However, significant results were only noted for the extracts of the plants *S. officinalis* and *V. locusta*. Another study [115] evaluated the efficacy of invasive plants as an alternative solution for preventing slug infestations. This study observed that plant materials of staghorn sumac (*Rhus typhina* L), giant goldenrod (*Solidago canadensis* L) and Japanese knotweed (*Fallopia japonica*) have strong antifeedant and barrier effects against Arionidae slugs. Among other products, Hollingsworth [21] reported that caffeine acts as a repellent against slugs and snails and, when applied on crops, caffeine leaves hardly have any phytotoxic effect on the crop. In another study, [115] conducted laboratory and semi-field experiments to evaluate the toxic, antifeedant and repellent efficacy of wood ash derived from seven plant species, sessile oak (*Quercus petraea*), European beech (*Fagus sylvatica*), European hornbeam (*Carpinus betulus*), silver fir (*Abies alba*), European spruce (*Picea abies*), common alder (*Alnus glutinosa*) and Spanish chestnut (*Castanea sativa*), and they observed the maximum mortality of slugs in oak and spruce wood ash treatments. Furthermore, wood ash from beech, oak, fir and spruce showed promising results as a physical repellent/barrier against *A. vulgaris*. Geochemical analysis of wood ash reveals a higher concentration of cobalt in beech, oak, fir and spruce wood ash [115]. However, Nechev [116] reported that a higher concentration of cobalt has a toxic effect on plants and animals. In addition, wood ash causes significant changes in soil texture, mineral compositions of soil, water retention capacity and pH, which reflect some challenges of using wood ash in a bio-rational control programme.

Lastly, a recent study [117] found evidence of asymmetrical interference competition between two geographically overlapping slug species in Ireland. The study suggested that *Geomalacus maculosus* Allman 1843 (Gastropoda: Arionidae, also known as the Kerry Slug), a non pestiferous and internationally protected slug species found in Western Ireland and North-Western Iberia [118], expresses molecules in its trail mucus that stimulate avoidance behaviour in a sympatric slug species (the tree slug *Lehmannia marginata*)—similar to ‘scent-marking’ in territorial mammals. It appears that the trail mucus exuded by *G. maculosus* contains molecules that are noxious to its native competitor, *L. marginata*, which strongly suggests that the mucus of this slug species may exhibit repellent properties. Preliminary molecular analyses of the mucus trails of each species suggested that—(1) the molecular properties of slug mucus vary greatly among species; and (2) some glycosidal molecules were present in *G. maculosus* mucus which were absent from the mucus of the sympatric *L. marginata*. However, further research is needed to establish whether the Kerry slug mucus also shows repellent properties against pestiferous slugs. Table 4 reports a list of natural products showing toxic, physiological, ovicidal, repellent and antifeedant effects against slugs.

## 3. Future Research Prospects

### 3.1. Nematodes as the Biocontrol Agent of Slugs

As shown in Table 1, a number of nematode species are slug parasites. However, so far, only *P. hermaphrodita* has been formulated as a commercial biocontrol agent with the trade names of Nemaslug^®^ (BASF) and SLUGTECH^®^ (Dudutech). Further research is needed to establish whether the other nematode species which possess slug control potential can be developed into commercial biocontrol agents. In-depth studies on each of these slug-parasitic nematodes should be conducted to evaluate their effect on nontarget organisms, the post-application survival rate and suitable production technology. Their pathogenicity should also be tested against a wide variety of slug species, including nonpestiferous native and protected gastropods, the understanding of which is particularly important in countries outside Europe, where restrictions have been imposed over the use of *P. hermaphrodita* [125].

On the other hand, as discussed, studies have cast doubts over the pathogenicity of the strain used in Nemaslug^®^ as the same strain has been cultured over a long period of time, highlighting the need for the identification of new strains of *P. hermaphrodita*. Surprisingly, little research has been carried out to identify novel (or wild) strains of *P. hermaphrodita* and to test their potential to control slugs. Recently, Cutler [37] examined the pathogenicity of nine wild strains of *P. hermaphrodita* found in the UK against the slug *D. invadens* and compared the results against the pathogenicity of the commercial strain of Nemaslug^®^. The study found the wild strains to be causing rapid mortality and feeding inhibition in *D. invadens* compared to the commercial strain. Thus, the isolation of new strains of *P. hermaphrodita* from different regions provides an exciting avenue for further research. In particular, with a better understanding of *P. hermaphrodita’s* wild strain bacteria–nematode association and their virulence on different slug species, their genetic study to recognise which genes are related with pathogenicity will help to develop these new strains of *P. hermaphrodita* as an efficient biocontrol agent of slugs.

Lastly, in order to identify new nematode species/strains with slug controlling potential, advanced methodologies should be adopted. Nematodes are microscopic organisms and their presence in the slug body cannot be detected by the naked eye in an early stage of infection. The current nematode identification method relies on the manual hand-picking of slug samples from the field, followed by dissection of the slug in the laboratory and isolation of nematodes from the slug body under a microscope. This process is labour-intensive and time-consuming. Thus, it is important to look for alternative screening approaches that can detect nematode infection in slugs rapidly. Multispectral imaging is one such method adopted in modern agriculture that has tremendous applications from identifying pests, disease and weeds, to optimising pesticide usage [126,127,128].

### 3.2. Sciomyzid Flies as the Biocontrol Agents of Slugs

The slug-preying potential of sciomyzid flies was noticed a long time ago [69,129]. However, unlike nematodes, comprehensive studies have not been performed on sciomyzid flies’ slug controlling potential, which has prevented their integration into biological slug control programmes. To date, most of the research on sciomyzids has focused on the control of snails which act as a vector of liver fluke and schistosomiasis diseases. We, therefore, propose the following future research avenues.

We suggest more research to be carried out on *T. elata’s* larval feeding behaviour, prey preference and functional response to different prey densities of slug species. Further, to date, research on the biocontrol potential of *T. elata* against slugs has been mostly conducted under laboratory conditions. Field trials will help validate the laboratory findings and will also allow the identification of nonprey food items that might be important in *T. elata* larval development that are currently unknown.

Moreover, the current understanding of their habitat preference is quite vague. The current thought is that most species of Sciomyzidae are wetland specialists and are regarded as bioindicator species of potential wetland. However, there are some species that confine themselves to terrestrial habitats. Williams [130] described Sciomyzidae residing in a wide range of habitats from terrestrial to freshwater, brackish water and maritime shoreline habitats. It was also assumed that the microhabitats in which sciomyzid larvae are available are selected by the ovipositing female, where larval survival is most likely to be successful. The questions that arise here is—if Sciomyzidae have such a broad habitat preference, why has their predation on slugs not been identified from different regions (see Table 2) There is anecdotal evidence that *T. elata* is in the decline around wide parts of both UK and Ireland. Therefore, we suggest that more in-depth research needs to be invested in exploring the habitat preferences of *T. elata* and other slug-controlling sciomyzid species. Understanding this could facilitate the construction of such habitats artificially, which could open the door for the commercialisation of this biocontrol agent.

Apart from *T. elata*, knowledge on the slug preying potential of other sciomyzid species is relatively unknown. Although nine sciomyzid species have shown slug-controlling behaviour [67,78], no in-depth investigation has been conducted to explore their feeding (larvae and adult), host and habitat preferences, and life tables. Comparison of the ecological behaviour of these five slug-killing sciomyzid species against that shown by *T. elata* will help identify more effective sciomyzid candidates for biocontrol programmes for slugs.

### 3.3. Carabid Beetles as the Biocontrol Agent of Slugs

It has been observed that most scientific studies have shown interest towards examining the slug control potential of mainly large-sized carabid beetles. In [89], it is argued that the medium-sized carabid beetles’ biocontrol potential against slugs and their final impact on reducing pest numbers and crop loss is yet to be fully examined. Although large-sized carabids are potential predators of slugs, medium-sized beetles such as *A. dorsale* and *A. fuliginosum* also control slugs and have been observed to cause a significant impact on the density of subsequent generations of slugs. Therefore, it is important not to overlook the potential of medium-sized carabid beetles as a predator of slugs in future research.

There has been much work on the use of Carabidae as bioindicators of sustainable agricultural practices and much investigation of their ecology in conservation headlands and field margins [131,132]. However, this work is rarely linked to the potential pest suppression benefits of Carabidae in a conservation biological control framework. A large challenge for the research community is to make the link from habitat management (conservation headlands, field margins and beetle banks) to landscape ecology of the beetles and subsequent pest suppression effects, and finally, to the agricultural productivity of arable farms.

A key factor affecting the efficacy of Carabidae as natural pest control agents of slugs is the timing of activity of pest slugs and their carabid predators. Reich [84] monitored the pest and predator phenology simultaneously with refuge and dry pitfall traps in grass crops in Oregon, USA. Similar studies accounting for phenology differences should be conducted across other valuable crops worldwide. Critically integrating phenology studies with gut content analysis and conservation biocontrol interventions in a landscape context should allow more targeted conservation biocontrol involving Carabidae to be undertaken.

### 3.4. Natural Products as Bio-Rational Control Agents of Slugs

As discussed, some research interest has been shown towards examining the toxic, ovicidal, repellent and antifeedant properties of essential oils and other natural products. Among these, essential oils are a promising and easily commercially available natural solution. Essential oils are promising because they can kill slugs, as well as boost plants’ defence, by enhancing their repellent, irritation and antifeedant properties against slugs. Prior studies in the context of other pests such as mites, coleopteran, mosquitoes and cockroaches show that essential oil-treated plants are able to demonstrate repellent, irritation and antifeedant effects towards these pest organisms [109,133,134]. Thus, essential oils might be able to offer all-round plant protection from slugs, which cannot be achieved using natural enemies. While the toxicity of some essential oils has been confirmed against some slugs, their repellent, irritant and antifeedant properties against slugs have hardly been studied and, therefore, needs further examination.

Overall, the research conducted on natural products addresses only fundamental questions and have mostly been carried out in laboratory conditions. Further in-depth and field studies are needed to recommend the inclusion of these natural products in IPM for slugs. For instance, future studies should conduct chemical analysis of the natural products to identify the active compounds of the natural products that are responsible for toxic, repellent or antifeedant properties. This will help develop commercial bio-pesticides in the long term. Currently, there is no commercial bio-repellent and/or bio-antifeedant available in the EUR 650 million global molluscicides market. In the short term, more detailed guidelines need to be provided regarding the use of natural products in the field. For instance, scientists must clarify particularly the following questions—how should the natural products be applied in the fields, i.e., on the plants or the ground? In what concentration should the natural products be applied? And how frequently do the natural products need to be applied for effective slug control? More field research is needed to address these questions.

Furthermore, from a conservation and biodiversity standpoint, the effect of the bio-rational products should also be tested on a wide range of nontarget organisms, including threatened and endangered species, which have largely been overlooked in the literature. Addressing this research gap is crucial to provide malacologists with a better understanding of the wider physiological and ecological effects of the bio-rational products without which the actual benefit of these bio-rational pest control measures will never be known. Lastly, scientific evidence on these biocontrol/bio-rational agents of slugs (nematodes, sciomyzid flies, carabid beetles and essential oils) reveal that, although they possess slug controlling properties, they have individual limitations. Therefore, on their own, these biocontrol/bio-rational agents, at the moment, are unlikely to be capable of fully controlling slugs. This concern makes a strong case for examining whether these biocontrol/bio-rational agents can be used in combination with control slug populations more effectively. Prior studies have shown that the combination of two or more control techniques may result in a ‘synergistic effect’ on pest organisms, i.e., the combined use of several control agents can result in better control of pests than the sum of the individual effects [135,136]. To date, no research has been undertaken to examine the presence of any synergistic or antagonistic effect between nematodes, carabid beetles, sciomyzid flies and essential oils. This could be an exciting avenue for future research because the comparison between the combined biocontrol/bio-rational potential of the four agents and individual slug control potential of each agent will help to provide a holistic understanding of how these biocontrol/bio-rational agents should be deployed in the field—i.e., alone or in combination (and in what combinations) for superior slug control.

## 4. Conclusions

Agricultural policymakers are under immense pressure to honour the Paris Climate Agreement and fulfil the sustainable development goals, and therefore, they are pushing scientists towards exploring novel and effective biological solutions for managing agricultural pests. An example of this is the proposed ban on the use of the chemical molluscicide metaldehyde in the UK from 2022. In 2018, the Farmers Weekly Periodical called the metaldehyde ban a ‘massive blow for growers’ because there is a limited number of alternative measures available for slug control that are environmentally benign and effective. This literature review shows that despite a significant amount of scientific interest invested towards identifying effective ‘natural’ or ‘biological’ measures for controlling slugs, there are only two bio-molluscicides commercially available in the form of nematode products. However, as discussed, there exist concerns over the effectiveness of these biocontrol agents. Therefore, we suggest that more urgent research is needed to improve the efficiency of these biocontrol agents, for example, by exploring novel ‘wild’ strains of *P. hermaphrodita* that are more effective in controlling slugs and formulating them into biocontrol agents. We also argue that it is important to look beyond *P. hermaphrodita* and conduct an in-depth investigation into the biocontrol potential of other nematodes that have slug control capabilities in order to formulate them into commercial biocontrol agents.

On the other hand, although the slug-control potential of sciomyzid flies and carabid beetles has been known for decades, there still exist a number of crucial research gaps which we have highlighted in this paper. For instance, research examining the slug control efficiency of *T. elata*, which is the most studied sciomyzid species concerning slugs, has been performed mainly in laboratory conditions. Thus, more field-based observations are needed without which the question remains—can *T. elata* be effective additions to practical biocontrol regimes for slugs? Similar to nematodes, we also suggest that it is important to look beyond *T. elata* and examine the biocontrol potential of other sciomyzid species and their habitat preferences. On the other hand, Carabidae are probably best as a conservation biocontrol agent, but there remains a research gap between their ecology in conservation headlands, field margins and beetle banks and their biocontrol potential in the arable fields.

Furthermore, we find that in the last few years, plant-derived essential oils have also attained importance in slug control, as recent studies have shown their molluscicide properties in managing terrestrial gastropods. While essential oils can be easily added to biocontrol programmes for slugs as they are commercially available, concerns lie with the side-effects from the application of essential oils, i.e., impact on nontarget organisms. Thus, we argue that more research should be directed towards examining and finding strategies to reduce such concerns. In addition, considering the individual concerns of slug-controlling nematodes, sciomyzid flies, carabid beetles and essential oils, it could also be worth investigating if there exist any synergistic effects between these natural enemies and products. Lastly, as it might be practically impossible to eradicate slugs completely from fields, it is also vital to conduct in-depth research on the natural products that can enhance plants’ defence against slugs, i.e., repellent, irritant and antifeedant effect against slugs.

Lastly, we urge scientists to pursue rigorous research on exploring biological measures to control other agricultural pests. The adoption of biological pest control strategies in agriculture will not only help manage pests and improve crop growth in an eco-friendly way, but also meet the customer’s demand for organic products. Furthermore, it will enable us to achieve the sustainable development goal of responsible consumption and production and reinforce our commitments towards the Paris Climate Agreement.

## Figures and Tables

**Table 1:** List of Phasmarhabditids that associate with slugs.

Nematode Species	Host Slug Species	Geographical Location	Reported by
*P. hermaphrodita*	*D. reticulatum*, *D. caruanae*, *D. invadens*, *A. distinctus*, *A. silvaticus*, *A. intermedius*, *A. ater*, *A. lusitanicus*, *T. sowerbyi*, *T. budapestensis*	UK	[17,23,37,38]
	*P. iberica* (Limacidae)	Iran	[51]
	*D. reticulatum*	USA	[33]
	*D. reticulatum*, *D. laeve* and *L. valentiana*	California	[34]
	*D. reticulatum*	New Zealand	[36]
	*D. reticulatum*	Chile	[52]
	*Lehmannia marginata*	Egypt	[17]
*P. neopapillosa*	*L. cinereoniger*	UK	[53]
	*D. reticulatum, D. panormitanum, A. ater, A. distinctus*	UK	[44]
*P. papillosa*	*D. panormitanum, D. reticulatum*	South Africa	[54]
	*A. vulgaris*	Slovenia	[42]
	*D. reticulatum*	USA	[35]
*P. californica*	*A. hortensis* agg., *D. reticulatum* and *L. valentiana*	USA	[35]
		New Zealand	[55]
	*G. maculosus* (non-pathogenic)	Europe	[56]
	*A.rufus*	Canada	[57]
*P. safricana*	*D. reticulatum*	South Africa	[49]
*P. apuliae*	*M. sowerbyi* and *M. gagates*	Italy	[47]
*P. tawfiki*	*L. flavus*	Egypt	[58]
*P. kenyaensis*	*P. robustum*	Kenya	[59]
*P. zhejiangensis*	*P. bilineatus Benson, PB*	China	[60]
*P. bonaquaense*	*M. tenellus*	Czech Republic	[47]
*P. bohemica*	*D. reticulatum*	Czech Republic	[61]

**Table 2:** List of slug-controlling sciomyzid species.

Sciomyzid Species	Host Slug	Geographical Location	Reported by
*Euthycera arcuate*	*Pallifera* spp., *Philomycus* spp.	North America	[6,67,76]
*Euthycera chaerophylli*	*Deroceras* spp.	Not specified	[67]
*Euthycera cribrata*	*D. reticulatum*	Not specified	[1]
*Euthycera stichospila*	Not specified	Not specified	[1]
*Limnia unguicornis*	Not specified	Not specified	[1]
*Tetanocera clara*	*P. morse, P. rafinesque*	USA (New York and Ohio)	[76]
*Tetanocera elata*	*D. reticulatum,D. invadens*, *Deroceras laeve*, *A. hortensis*, *A. fasciatus*, *A. intermedius*, *L. flavus*, *M. tenellus*, *T. budapestensi*, *T. sowerbyi*, *G. maculosus* (protected slug species)	Ireland	[15,19,77,79]
*Tetanocera plebeja*	*D.slaeve, D. reticulatum*	USA (Ohio)	[78]
*Tetanocera valida*	*Deroceras* spp.	North America	[76]

**Table 3:** List of slug-controlling carabid species.

Carabid Species	Host Slug	Geographical Location	Reported by
*Abax ater*	*A. subfuscus*, *A. intermedius*, *A. circumscriptus*, *A. rufus*, *L. tenellus*	Germany	[98]
*Abax parallelepipedus*	*D. reticulatum*, Arion spp.	Norway	[80,89]
*Abax parallelus*	Not specified	Not specified	[89]
*Agonum muelleri*	Not specified	USA	[84]
*Amara aulica*	Not specified	Norway	[89]
*Amara lunicolis*	Not specified	Norway	[89]
*Amara similata*	Not specified	Norway	[89]
*Calosoma frigidum*	*A. ater*	Not specified	[89]
*Carabus nemoralis*	*A. lusitanicus*, *A. ater*, *D. reticulatum*	Norway, UK	[81,89]
*Carabus granulatus*	*A. lusitanicus*, *D. reticulatum*	Austria	[89]
*Carabus problematicus*	Not specified	Europe	[88]
*Carabus violaceus*	*A. fasciatus*, *D. reticulatum*	Norway, UK	[81,89]
*Pterostichus madidus*	*D. reticulatum*	UK	[80]
*Pterostichus melanarius*	*A. lusitanicus (eggs)*, *D. retuculatum*, *A. distinctus*, *A. subfuscus*, *L. marginate*, *M. tenellus*, *T. budapestensis*	Norway, UK, Czech Republic	[89,99]
*Pterostichus niger*	*A. lusitanicus*, *Arion fasciatus*, *D. reticulatum*	Ireland, Finland, Norway, UK	[81,89,100,101]
*Pterostichus aethiops*	Not specified	Not specified	[89]
*Cychrus attenuatus*	Not specified	Not specified	[89]
*Cychrus caraboides*	*D. reticulatum*, *A. fasciatus*, *A. vulgaris* (eggs/juveniles)	UK, Finland	[81,100]
*Cyclotrachelus alternas*	*Not specified*	Not specified	[89]
*Diplocheila striatopunctata*	*A. ater*	Not specified	[89]
*Harpalus aeneus*	*D.reticulatum*	UK	[88]
*Harpalus latus*	Not specified	Norway	[89]
*Harpalus rufipes*	*D. reticulatum* (eggs)	Spain	[96]
*Megadromus antarcticus*	*D. panormitanum, D.reticulatum*	New Zealand	[102]
*Nebria brevicollis*	*D. reticulatum*	UK, USA	[84,88]
*Poecilus cupreus*	*A. lusitanicus*, *D. reticulatum*	Switzerland	[95]
*Poecilus laetulus*	Not specified	USA	[103]
*Poecilus lucublandus*	Not specified	Not specified	[89]
*Poecilus nigrita*	Not specified	Norway	[89]
*Poecilus oblongopunctatus*	Not specified	Norway	[89]
*Scaphinotus marginatus*	*D. reticulatum*	Not specified	[87]
*Scarites anthracinus*	*D.reticulatum*	Argentina	[104]

**Table 4:** List of natural products showing toxic, physiological, ovicidal, repellent and antifeedant effects against slugs.

Product Class	Product Names	Effect	Target Slug	Reported By
Essential Oils	Thyme, spearmint, pine, peppermint, garlic, rosemary, lemongrass and cinnamon oil	Toxic effect	*D. reticulatum*	[22]
	Sweet wormwood oil	Toxic and physiological effect	*Agriolimax agrestis*	[119]
	Neem oil	Ovicidal effect	*D. reticulatum*	[120]
	Birch tar oil	Repellent effect	*A. lusitanicus*	[114]
	Myrrhs oil *(Commiphora molmol and Commiphora guidotti)*	Repellent effect	*D. reticulatum*	[113]
	Carvone (natural compound present in caraway seed oils)	Antifeedant effect	*A. lusitanicus*	[121]
Plant Extracts	*Geranium robertianum*, *Lepidium sativum*, *Origanum vulgare*, *Salvia officinalis*, *Salvia pratensis*, *Saponaha officinalis*, *Thymus vulgaris*, *Trifolium repens* and *Valerianella locusta*	Antifeedant effect	*A. lusitanicus*	[20]
	Invasive Plant species extract (Japanese knotweed, Bohemian knotweed, Canadian goldenrod, Giant goldenrod, Staghorn sumac, Tree of heaven False indigo)	Toxic, repellent and antifeedant effect	*A. vulgaris*, *Arion rufus*	[115]
	Saponin-rich plant extracts (*Camellia oleifera*, *Gleditsia amorphoides* and *Quillaja saponaria)*	Toxic and antifeedant effect	*D.reticulatum*	[122]
Microbes	Fungal extract	Antifeedant effect	*A. vulgaris*	[123]
	*Verticillium chlamydosporium*	Ovicidal effect	*Not mentioned*	[32]
	Arthrobotrys was found to be parasitising the eggs of the slug *D. reticulatum*	Ovicidal effect	*D. reticulatum*	[32]
Others	Caffeine	Toxic effect, repellent effect	*D. reticulatum*, *Veronicella cubensis*	[21,22]
	Hydrated lime	Toxic and repellent effect	*Arion* sp.	[124]
	Plant wood ash	Toxic, antifeedant and repellent effect	*A. vulgaris*	[115]
	Mucus of Kerry slugs	Repellent effect	*L. marginata*	[117]
